# A Bayesian Approach for Remote Depth Estimation of Buried Low-Level Radioactive Waste with a NaI(Tl) Detector

**DOI:** 10.3390/s19245365

**Published:** 2019-12-05

**Authors:** Jinhwan Kim, Kyung Taek Lim, Kyeongjin Park, Gyuseong Cho

**Affiliations:** Department of Nuclear & Quantum Engineering, Korea Advanced Institute of Science and Technology, 291, Daehak-ro, Yuseong-gu, Daejeon 34141, Korea; kjhwan0205@kaist.ac.kr (J.K.); kl2548@kaist.ac.kr (K.T.L.); myesens@kaist.ac.kr (K.P.)

**Keywords:** remote-depth profiling, gamma spectral analysis, Bayesian inference, uncertainty estimation, radioactive nuclear waste, radiological characterization, nuclear decommissioning, radiation detection, low-resolution detector

## Abstract

This study reports on the implementation of Bayesian inference to improve the estimation of remote-depth profiling for low-level radioactive contaminants with a low-resolution NaI(Tl) detector. In particular, we demonstrate that this approach offers results that are more reliable because it provides a mean value with a 95% credible interval by determining the probability distributions of the burial depth and activity of a radioisotope in a single measurement. To evaluate the proposed method, the simulation was compared with experimental measurements. The simulation showed that the proposed method was able to detect the depth of a Cs-137 point source buried below 60 cm in sand, with a 95% credible interval. The experiment also showed that the maximum detectable depths for weakly active 0.94-μCi Cs-137 and 0.69-μCi Co-60 sources buried in sand was 21 cm, providing an improved performance compared to existing methods. In addition, the maximum detectable depths hardly degraded, even with a reduced acquisition time of less than 60 s or with gain-shift effects; therefore, the proposed method is appropriate for the accurate and rapid non-intrusive localization of buried low-level radioactive contaminants during in situ measurement.

## 1. Introduction

During the life cycle of nuclear facilities, a significant amount of radioactive waste is generated, resulting in large-scale land and building contamination. Characterization of these wastes is critical for decommissioning those contaminated sites because it can provide essential information related to design specifications and project planning required for environmental restoration [1,2,3]. In particular, acquiring knowledge of the depth profiling of radioactive contaminants is critical for choosing the most cost-effective decommissioning strategy because removing surface contamination at varying depths can significantly reduce the total disposal volume [4]. However, depth profiling remains challenging because porous materials, such as the soil and concrete that entrain the contaminants, also act as shielding materials. In fact, examples of wastes commonly encountered during the decommissioning of nuclear facilities include wastes buried inside such porous materials [5,6,7].

Traditional destructive methods, such as logging and core sampling, have been used for depth estimation; however, they are expensive and time-consuming [7,8]. Thus, various non-destructive techniques have been developed for remote-depth profiling including the relative attenuation method [9,10], principal component analysis (PCA) [11,12,13], and the approximate three-dimensional linear-attenuation method [14,15,16]. The relative attenuation method takes the relative difference in the attenuations of two primary peaks (i.e., the 32-keV X-ray and 662-keV gamma-ray peaks of Cs-137) in a measured spectrum to find the depth profile. However, the use of X-rays limits not only the maximum detectable depth to less than 2 cm due to their high attenuation, but also the number of specific radioactive sources. On the other hand, the PCA method extracts two principal component coefficients related to the depth of the buried radioactive source from a set of previously measured spectra with different burial depths. The synthetic angle is then defined based on the extracted coefficients to estimate the source depth. However, this method cannot effectively estimate the depth of a source buried more than 5 cm beneath the surface. Finally, the approximate three-dimensional linear attenuation method employs the well-known linear attenuation model [17] in 3D coordinates combined with information obtained from multiple measurements of gamma ray intensities on the surface of the material in which the radioactive contaminant is buried. This approach shows a clear improvement over earlier methods in terms of the maximum detectable depth up to 12 cm in sand for a 8.89-μCi Cs-137 source. Nonetheless, the aforementioned methods provide point estimates for the penetration depth of radioactive contaminants. That is, they ignore the uncertainty invariably associated with statistical fluctuations arising from physical processes that can only be determined by performing tedious repetitive measurements. In addition, the maximum detectable depth of existing methods is insufficient to detect significant amounts of internal contamination buried deep within a substance [5]. Likewise, gain-shift effects can degrade the performance of the existing methods because almost all detector-based systems are sensitive to changes in ambient temperature.

Therefore, we propose an advanced remote-depth estimation method for measuring buried radioactive contamination using Bayesian inference. Both simulation and experimental testing were conducted using a low-resolution NaI(Tl) detector to evaluate the performance of the proposed method under many possible scenarios. In addition, this work also emphasizes the influence of data-acquisition time and gain shifts upon the depth-estimation process. Lastly, we evaluated the sensitivity of the proposed model in terms of the prior distributions. 

## 2. Materials and Methods

### 2.1. Bayesian Inference

In statistical inference, there are two different approaches to probability interpretations, namely frequentist inference and Bayesian inference [18]. The frequentist inference is based on the idea that probability is equal to the expected frequency of occurrence over a long period. In this case, the frequentist assigns unknown parameters to fixed values. Thus, the frequentist inference does not allow probability statements about the parameters of a statistical process. For instance, the fact that a 95% confidence interval for the normal mean value is within a certain range does not mean that 95% of this confidence interval contains the true value [18]. Instead, what it means is that there is a 95% chance that, when computing confidence intervals repeatedly many times, the true mean would lie in the 95% confidence interval. By contrast, Bayesian inference is based on the idea that probability represents the degree of belief in an event. That is, this concept allows us to treat the parameters as random variables. In this sense, a Bayesian statistician would say that there is a 95% probability that the true value will fall within the 95% credible interval of the given data range. 

The objective of Bayesian inference is to determine the posterior distribution p(θ|y) of random variables θ given prior distributions p(θ) and likelihood function p(y|θ) . This consideration is well-represented in Bayes’ theorem [19]:

(1) $$p\left(\theta |y\right)=\frac{p\left(y|\theta \right)\;p\left(\theta \right)}{p\left(y\right)},$$

where p(θ) is the probability in our belief of θ without observation of the data y; p(y) is an evidence or normalization constant, from which the probability of the data is determined by integrating all possible values of θ ; and p(y|θ) is the probability of observing y generated by a model with θ. In fact, p(θ|y) is the refined probability of our belief in θ once y has been taken into account. 

The difficulty in applying Bayesian inference to many practical applications arises when the intractable high-dimensional integrals of the evidence must be computed. However, recent advances in computation and in marginal estimation techniques using variational inference make it possible to solve such problems. The underlying idea behind variational inference is to convert the computation of the posterior distribution into an optimization problem. First, a parameterized family of distributions q(θ;υ) (or equivalently, a variational distribution) is postulated. Then, we find the member of that particular family that minimizes the Kullback–Leibler (KL) divergence [19] of the exact posterior distribution. In fact, the KL divergence measures the closeness of the two distributions:

$$KL\left(q\left(\theta ;\upsilon \right)||p\left(\theta |y\right)\right)=\;E_{q}\left[\log\frac{q\left(\theta ;\upsilon \right)}{p\left(\theta |y\right)}\right]$$

(2) $$\;=\;-\left(E_{q}\left[\log p\left(\theta ,y\right)\right]-E_{q}\left[\log q\left(\theta ;\upsilon \right)\right]\right)+\log p\left(y\right),$$

where p(y) is intractable but has a constant value. Thus, minimizing the KL divergence is now equivalent to maximizing the evidence lower bound (ELBO):

(3) $$ℒ\left(\upsilon \right)=E_{q}\left[\log p\left(\theta ,y\right)\right]-E_{q}\left[\log q\left(\theta ;\upsilon \right)\right].$$

To simplify the inference further, a mean-field approximation can be assumed, where the parameters can be fully factorized into independent parts:

(4) $$q\left(\theta_{1},\;.\;.\;.\;,\;\theta_{n}\right)=\overset{N}{\underset{i=1}{\prod }}q_{i}\left(\theta_{i}\right).$$

Despite efforts at simplifying via mean-field approximation in the variational inference, model-specific derivations and implementations are still required, making the process rather complex. Automatic differentiation variational inference (ADVI) [20] can solve this problem by offering an algorithm for automatic solutions associated with variational inference. ADVI begins by transforming p(θ,y) into the unconstrained real-valued random variables p(θ,ζ). This transformation removes all original constraints on the latent variables, allowing us to consider the Gaussian distribution. Then, ADVI recasts the gradient of the variational objective function as an expectation over q In this way, one can make use of Monte Carlo methods to approximate $\nabla_{\theta }\log\left(\theta ,\;y\right)$. Further re-parameterization of the gradient in terms of a standard Gaussian is performed for the purpose of achieving efficient computation of the Monte Carlo approximations. Finally, ADVI uses a stochastic gradient optimization approach for the variational distribution. We implemented ADVI in the Python language together with the probabilistic programming framework of PyMC3 [21]. This powerful library allowed us to easily specify a probability model and then perform variational inference.

### 2.2. Likelihood Function and Priors

In order to compute the posterior distribution of the depth of a buried radioactive source, we begin by defining a mathematical model that describes an observed spectrum in terms of the burial depth, activity, and relative shift magnitude of the spectrum (and hence, the shift). The spectrum measured from a collimated detector can be calculated as follows:

(5) $$M_{i}=N_{0}\delta \varepsilon =AP\delta \varepsilon =\;\frac{AP\delta }{4\pi h^{2}}e^{-\mu_{A}h}f\left(z,\;\eta i\right)+cB_{i}\;\mathrm{for}\;i=1,\ldots ,K,$$

where $M_{i}\;$is the measured spectrum (s^−1^); i is the channel or energy index (0<i≤K) ; $N_{0}\;$is the total intensity of gamma rays originally emitted from the source (s^−1^); δ is the effective front area of the detector (cm^2^); ε is the correction coefficient due to the attenuation in a material, gain-shift effects caused by temperature, and the inverse-square law; A is the activity in the radioisotope of interest per decay (μCi); P is the total gamma emission probability (i.e., 2 γs^−1^ Bq^−1^ for the 1173- and 1333-keV gamma rays of Co-60); $\mu_{A}$ is the linear attenuation coefficient of gamma rays in air (cm^−1^); h is the detection height between the detector and the surface of a material (cm); z is the buried depth of a radioactive source (0≤z≤D cm) in a section of material from the front surface; η is the relative magnitude of the shift in the spectrum; $B_{i}$ is the background spectrum in the measurement environment with K channels; c is its proportionality constant; and f(z, ηi) is the function for bilinear interpolation. To compute f(z, ηi) the K×D response matrix (or equivalently, the reference spectra), for a radioisotope should be determined by measuring the spectra at different depths ranging from 0 to D cm with certain intervals. It should be noted that all spectra obtained for the response matrix were normalized to the total count of the spectrum measured at a depth of 0 cm. Consequently, f(z, ηi) can be calculated as follows:

(6) $$f\left(z,\;\eta i\right)=\frac{1}{\left(z_{H}-z_{L}\right)\left(i_{H}-i_{L}\right)}\left[z_{H}-z\;z-z_{L}\right]\left[\begin{array}{c} f\left(z_{L},i_{L}\right)\;f\left(z_{L},i_{H}\right)\\ f\left(z_{H},i_{L}\right)\;f\left(z_{H},i_{H}\right) \end{array}\right]\left[\begin{array}{c} i_{H}-i\\ i-i_{L} \end{array}\right].\;$$

**Figure 1:**
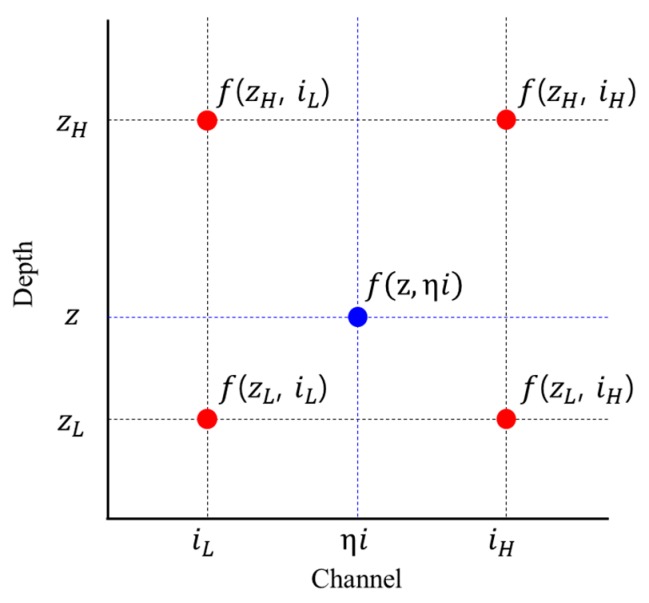
Example of bilinear interpolation for function *f* on target point (*z*,*ηi*) in a rectangular constellation formed by four grid points $(f\left(z_{L},i_{L}\right),\;f\left(z_{H},i_{L}\right),\;f\left(z_{L},i_{H}\right),\;\mathrm{and}\;f\left(z_{H},i_{H}\right))$.

As shown in Figure 1, f(z, ηi) is the interpolation point. In addition, $f\left(z_{L},i_{L}\right)$, $f\left(z_{H},i_{L}\right)$, $f\left(z_{L},i_{H}\right)$, and $f\left(z_{H},i_{H}\right)\;$are the closest points to the f(z, ηi) among the known points from the K×D response matrix. In other words, the spectrum of 512 channels with the buried depth of the source at z cm and the shifted magnitude of η in the spectrum can be expressed as:

(7) $$M=\frac{AP\delta }{4\pi h^{2}}\left[f\left(z,\;\eta \right),\;f\left(z,\;2\eta \right),\ldots ,\;f\left(z,\;512\eta \right)\right]+cB.$$

Here, the value *δ* can be calculated from a laboratory experiment by placing a radioactive source on the surface of the material (at 0 cm in depth), and can be expressed as:

(8) $$\delta =\frac{4\pi r^{2}N}{APe^{-\mu_{A}r}},\;$$

where N is the total net counts in the spectrum (s^−1^) and *r* is the detection height (cm).

As a matter of fact, our model prescribes the function f(z,A, η, c). Nonetheless, the measured spectrum in practice experiences inevitable interference from the presence of irreducible uncertainties arising from physical processes, such as radioactive disintegration. Thus, the spectrum can be assumed to have a normal distribution with a zero mean and variance of $\sigma^{2}$:

(9) $$M\;\sim \;f\left(z,A,\;\eta ,\;c\right)+N\left(0,\;\sigma^{2}\right),$$

(10) $$\;P\left(M|z,A,\;\eta ,\;c\right)=N\left(f\left(z,A,\;\eta ,\;c\right),\;\sigma^{2}\right).\;$$

When the initial information about the distributions of z,A, η, c, and $\sigma^{2}$ is available, it should be included as the priors. Table 1 shows the priors prescribed by the model where *A*, *c*, and $\sigma^{2}$ should be continuous and positive-only, and the gamma distribution can therefore be selected accordingly. However, one may wonder how the parameters were determined, that is, a shape parameter α and an inverse scale parameter β in the gamma distribution, because the parameters represent our degree of belief. In this work, we divided the measured spectrum by the acquisition time and analyzed them on a one-second basis, which was true in case of the background spectrum. In this regard, the most probable value of c would be one. Therefore, the determination of the parameters with gamma(1, 1), i.e., the mean and variance are equal to one, would be reasonable. In addition, the parameters can be considered as being equal to gamma(1, 1) since $\sigma^{2}$ is expected to yield a small value. Although the choice on the parameters in the gamma distribution for A could be somewhat ambiguous, it would still be appropriate to determine the parameters with gamma(1, 1); this was because our belief is that the level of the source activity would be low. On a contrary, the parameters *z* and η should be confined within certain ranges. In fact, the range for *z* was selected from 0 to *D* cm, which was the maximum burial depth of the radioactive contaminant to be considered; the value *D* was selected as 50 cm (or 60 cm) such that the proposed method would search extensively for the burial depths of the source ranging from 0 to 50 cm. As for the parameter η, the value was chosen from a range of 0.85 and 1.15 in consideration of the relative Cs-137 peak positions in the NaI(Tl) spectra depending on the temperature ranging from 0 to 50 °C such that the selected range would contain all possible values for the shift magnitude [22]. It is worth emphasizing that any values for the parameters (i.e., α and β) in which their multiplication would be equal to one or other choices that would yield a small mean value in the gamma distribution is possible. In addition, other distributions, such as a truncated normal distribution and triangular distribution, could be selected based on knowledge of the evaluators. Since the selection on prior distributions could influence posterior distributions, the sensitivity analysis on prior distributions was necessary to verify the robustness of our model, which is presented in Section 3.5.

### 2.3. Monte Carlo Modeling and Simulation

**Figure 2:**
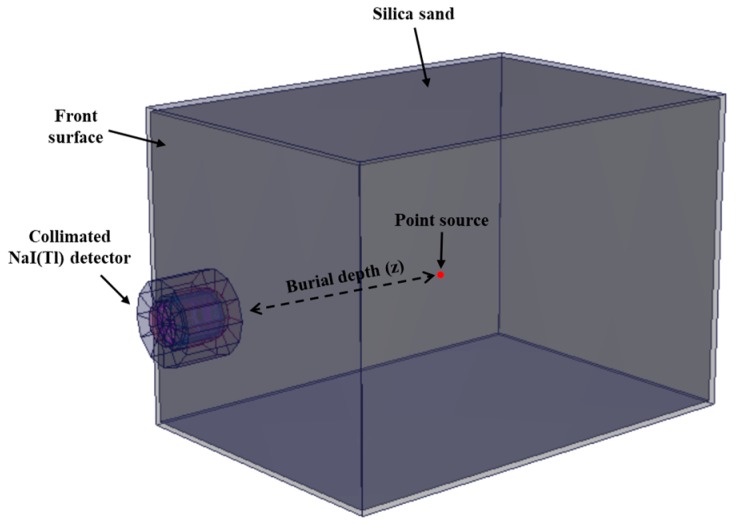
Schematic of the geometry defined for MCNP6 simulation. A collimated NaI(Tl) detector was placed 6 cm away from the front surface and a radioactive Cs-137 source was located inside silica sand.

In order to validate the proposed method, we performed Monte Carlo modeling and simulations using Monte Carlo N-Particle Transport Code, version 6 (MCNP6) [23]. A schematic of the MCNP6 model used for the simulation is reported in Figure 2. The simulation model consists of a NaI(TI) detector located 6 cm away from the surface of a box that is filled with sand of density 1.7 g cm^−3^ [24]. The detector itself is surrounded by a hollow cylindrical lead shield lined with copper for minimizing the scattered gamma rays and X-ray fluorescence from lead. A pulse-height tally was used to simulate the gamma-ray spectra. In addition, an FT8 Gaussian-energy-broadening card was applied to mimic the physical spectra as realistically as possible. In fact, this feature simulates the peak-broadening effect arising from a physical radiation detector based on coefficients “a,” “b,” and “c.” These coefficients allow the MCNP6 to recognize the continuous values of the full width at half maximum (FWHM) in the energy range of interest based on the non-linear function in Equation (11), where *E* is the incident gamma-ray energy. We employed parametric optimization using a genetic algorithm to find the optimal value of these coefficients [25]:

(11) $$\mathrm{FWHM}=a\;+b\sqrt{E\;+cE^{2}}.$$

The radioisotope Cs-137, which is one of the most predominant anthropogenic radioactive contaminants, arising as it does in fission fragments from spent nuclear fuel, was used and treated as a point source during the simulation. To obtain the response matrix, the point source was placed in sand at depths of 0, 1, 3, 5, 7, 10, 15, 20, 25, 30, 40, 50, and 60 cm. At each depth, a total of 3 × 10^9^ particles were generated to achieve a sufficient counting statistic in the simulated spectra. For the test spectra, the point source was buried at a depth from 0 to 60 cm with 3-cm intervals. In this case, a total of 2 × 10^8^ gamma particles were transported at each depth.

### 2.4. Experimental Setup

**Figure 3:**
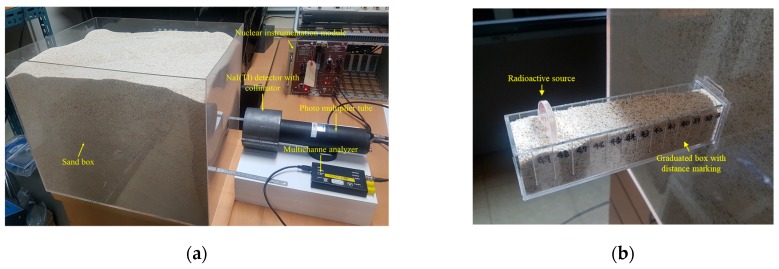
(**a**) Experimental setup for the gamma-ray measurement and (**b**) a graduated box for adjusting the burial depth of a radioactive source.

The experimental setup for the acquisition of gamma-ray spectra is shown in Figure 3a. The setup was composed of a sandbox filled with fine silica sand in which a radioactive source was buried and the two-inch NaI(Tl) detector was located 6 cm away from the box surface. The dimensions of the box were 50 cm × 40 cm × 40 cm (length × width × height), and it was constructed from acrylic sheets of thickness 0.3 cm. The use of acrylic and its thickness were chosen to allow almost all gamma rays to scatter exclusively within the sand matrix. In the experiment, a sealed Co-60 source was used in addition to Cs-137, because Co-60 is also found in nuclear environments resulting from the neutron activation of steel parts. The activity of the Cs-137 and Co-60 sources were 0.94 μCi and 0.69 μCi, respectively. The source was buried in a graduated box (50 cm × 3 cm × 3 cm) filled with sand, as shown in Figure 3b. Then, the box was inserted into the sandbox such that the exact distance of the source from the front of the sandbox would be achievable. The detector was placed inside a cylindrical lead collimator with a thickness of 2 cm, clad in a 0.5-cm-thick copper layer.

In the experiment, the spectra for the response matrix and the test set were measured at the same position of each radioactive source, as mentioned in Section 2.3, up to depths of 50 cm and 48 cm, respectively. The spectra for the response matrix were measured for 50 min to minimize statistical fluctuations in the spectra, while the test spectra were measured for 10 min. In addition, a background spectrum for the response matrix was also acquired under the same condition but without sources. Furthermore, the energy ranges of the spectra for Cs-137 and Co-60 were chosen from 300 to 1500 keV (i.e., 369 channels) and 700 to 1500 keV (i.e., 246 channels), respectively. During the experiment, energy calibration was not strictly performed because the proposed model was capable of compensating for gain-shift effects.

## 3. Results

### 3.1. Depth Estimation of the Buried Cs-137 Based on Simulated Spectra

**Figure 4:**
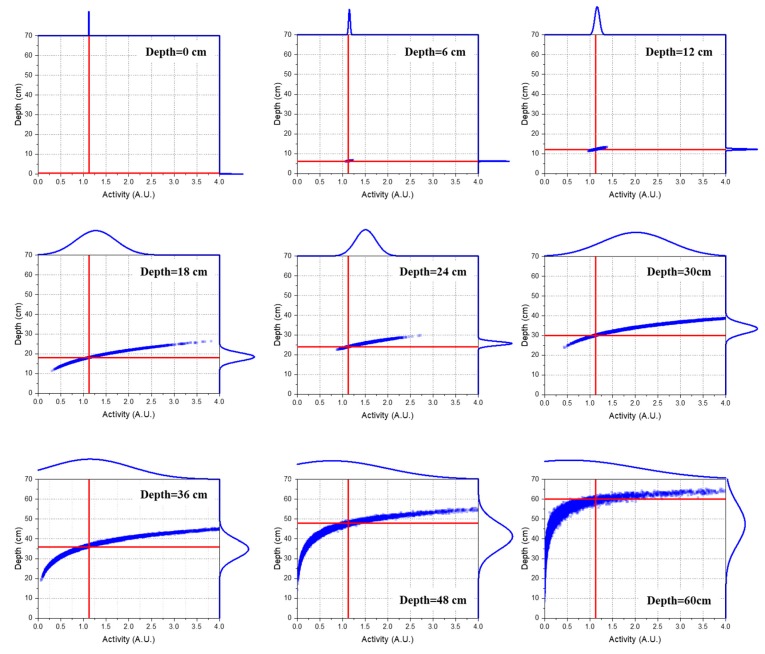
Joint probability distributions between the depth and activity of Cs-137 for selected depths simulated via MCNP6. The scatter dots in the central area depict the correlations between the estimated depth and activity with red lines representing true values, while the curves outside the plot area represent their corresponding densities.

Figure 4 shows the joint probability distributions of the depth and activity of Cs-137 sources for selected depths, where the red line represents the true values for the depths and activities. As expected, the joint distribution gradually spread out with a strong positive correlation with increasing source depth. This was mainly due to the increased number of gamma rays scattering in the sand matrix and the decreased number of detected photons, which consequently caused an increase in the statistical fluctuations in the spectra. Moreover, the positive correlation was clear because the activity was inversely related to the square of the depth (see Equation (5)). In this regard, the estimated values of the activity changed more sensitively when the source was deeply buried.

**Figure 5:**
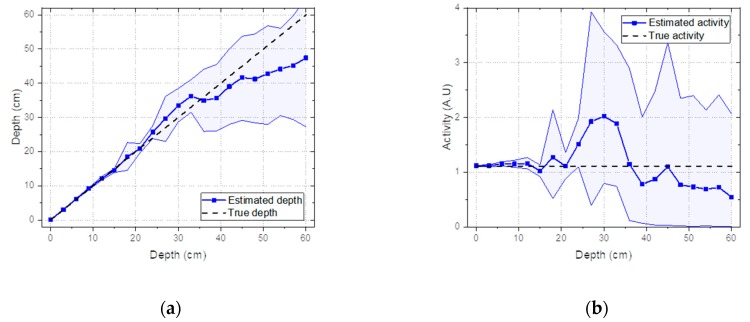
(**a**) Estimated depth and (**b**) activity with a 95% credible interval for spectra simulated with Cs-137 buried in sand from 0 to 48 cm with 3-cm intervals.

The estimated depth with a 95% credible interval for the Cs-137 source buried in sand over a range from 0 to 60 cm with 3-cm intervals is shown in Figure 5a. It shows that the true depth was well-approximated by the mean value of the estimated depth up to 45 cm. Beyond this depth, the estimated values tended to be underestimated. Nonetheless, all true depths fell within the 95% credible interval of the estimated depths until reaching a depth of 60 cm. In fact, the values for the source depths used to generate the response matrix rarely included those used to generate the test spectra. In other words, the interpolation method estimated the spectra that were not predetermined at certain depths well and therefore it was not necessary to take measurements for the response matrix at every single depth. As shown in Figure 5b, the estimated activities also closely agreed with the true activities, considering the 95% credible intervals. However, it is worth noting that the credible interval of the activities was more susceptible to fluctuation due to the inverse-square law.

### 3.2. Depth Estimation of Buried Cs-137 and Co-60 Based on Experimental Spectra

**Figure 6:**
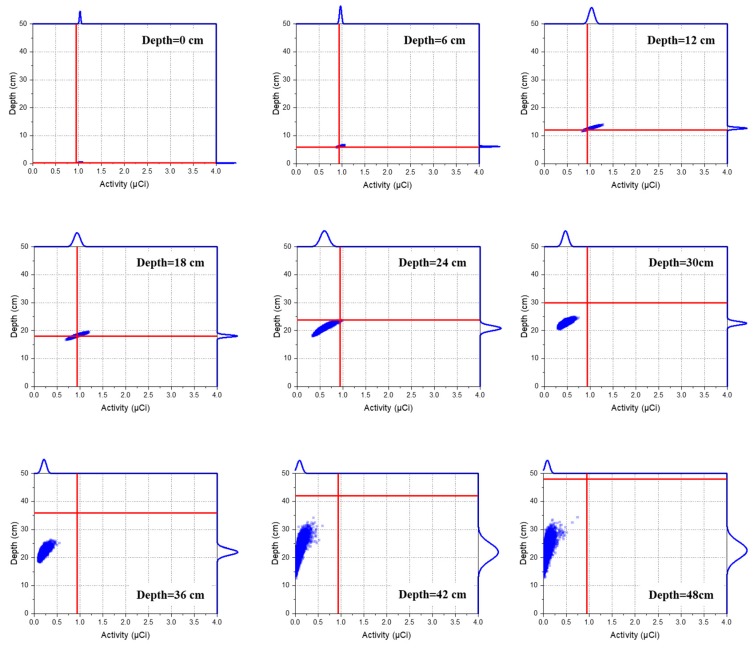
Joint probability distributions between the depth and activity of Cs-137 for selected depths, as measured experimentally. The scatter dots in the central area depict the correlations of estimated depth and activity, with red lines representing true values, while the curves outside the plot area represent their corresponding densities.

**Figure 7:**
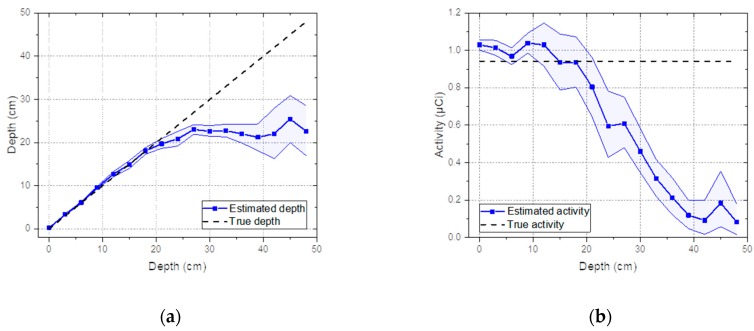
(**a**) Estimated depth and (**b**) activity with a 95% credible interval for spectra measured from Cs-137 buried in sand from 0 to 48 cm with 3-cm intervals.

**Figure 8:**
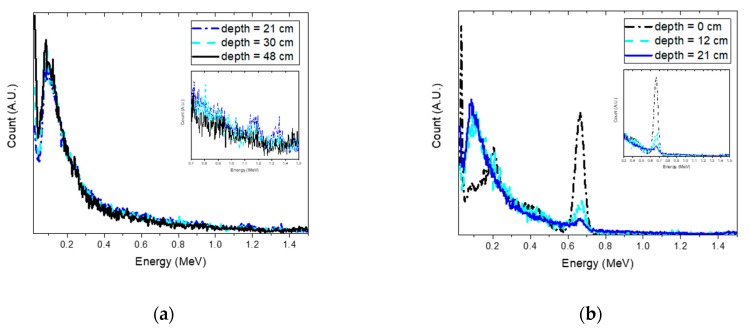
Normalized spectra for different depths of Cs-137 with a 600-s acquisition time: (**a**) the source was buried at depths of 21 cm, 30 cm, and 48 cm; and (**b**) the source was buried at depths of 0 cm, 12 cm, and 21 cm. The spectra were normalized to the total count across all energies for comparison purposes only. The inset shows the spectra within the energy range used for analysis.

The estimation of the joint probability distributions of the depth and activity of Cs-137 for selected depths is reported in Figure 6. From the plots, we can see a similar trend to the simulation results. In particular, this can be clearly seen in Figure 7a, in which the true depth was well-approximated by the estimated depth up to 21 cm. Beyond this, the value of the estimated depth began to be underestimated and to fluctuate. It is interesting to note that this depth was much lower than the value obtained using simulations. This was because the influence due to the attenuation and background became significant with increasing source depth, resulting in no remarkable feature differences in the acquired spectra, as illustrated in Figure 8a. In addition, the weak activity of the Cs-137 source used for the experiment could also be attributed to the obtained result. By contrast, noticeable feature differences were observed in the spectra at depths from 0 to 21 cm, as shown in Figure 8b. Based on the results, the maximum detectable depth of Cs-137 for the experimental setup was determined to be 21 cm.

**Figure 9:**
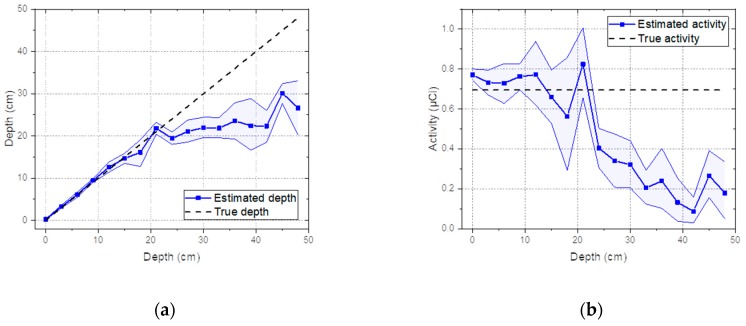
(**a**) Estimated depth and (**b**) activity with a 95% credible interval for spectra measured from Co-60 buried in sand from 0 to 48 cm with 3-cm intervals.

**Figure 10:**
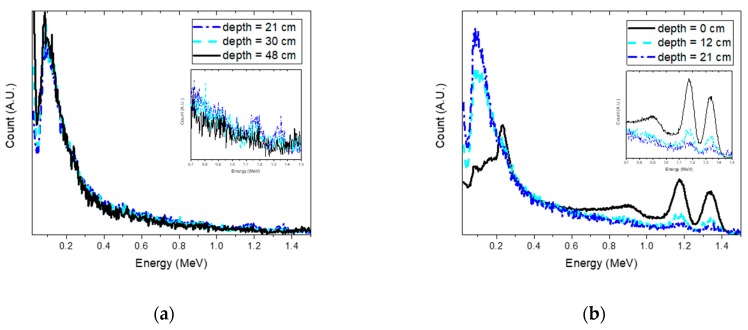
Normalized spectra for different depths of Co-60 with a 600-s acquisition time: (**a**) the source was buried at depths of 21 cm, 30 cm, and 48 cm; and (**b**) the source was buried at depths of 0 cm, 12 cm, and 21 cm. The spectra were normalized to the total count across all energies for comparison purposes only. The inset shows the spectra within the energy range used for analysis.

The same experiment was also performed with the Co-60 source. Figure 9a shows the estimated depth with a 95% credible interval for the Co-60 source buried in sand from 0 to 48 cm with 3-cm intervals. As expected, the same trend was observed as in the Cs-137 case; the true depth was well-approximated by the mean value of the estimated depth up to 21 cm and the estimated depth gradually deviated from the true depth and fluctuated beyond the depth of 21 cm. The reason for this was the same as that mentioned earlier, namely differences in spectral features became negligible with increasing source depth, as shown in Figure 10.

### 3.3. Effect of Acquisition Time

**Figure 11:**
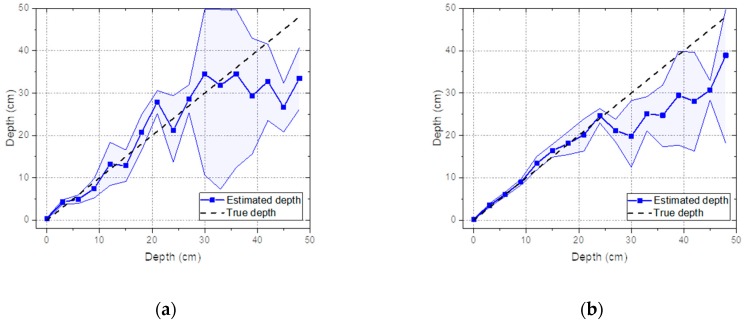
Estimated depth with a 95% credible interval analyzed for Cs-137 buried in sand over the range of 0 to 48 cm at 3-cm intervals from the experiment and the corresponding value of the true depth: (**a**) acquisition time of 10 s and (**b**) acquisition time of 60 s.

**Figure 12:**
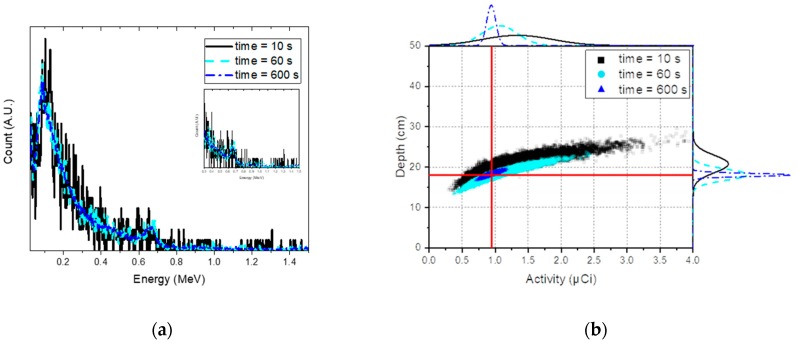
(**a**) Normalized spectra for Cs-137 buried at depths of 18 cm with acquisition times of 10 s (black solid line), 60 s (blue-sky dashed line), and 600 s (blue dash-dotted line). (**b**) Joint probability distributions between the depth and activity analyzed for the same spectra; the red line represents the true value of the depth and activity. The inset shows the spectra within the energy range used for analysis.

To perform an in-depth analysis of the sensitivity with respect to acquisition time, gamma-ray spectra with reduced acquisition times were analyzed at the same varying depths as those mentioned in Section 3.2. Figure 11a,b shows the estimated depths with 95% credible intervals analyzed for the spectra of Cs-137 with acquisition times of 10 s and 60 s, respectively. Despite the very short acquisition time, an identical trend was observed as in the spectra obtained over 600 s; the true depths were well-approximated for the mean values of the estimated depths up to 21 cm, which was the maximum detectable depth found in Section 3.1. Surprisingly, even at depths beyond the maximum detectable depth, the true depths seemed to yield a better approximation using the estimated depths with consideration of the 95% credible intervals. This may have been due to the statistical fluctuation in spectra caused by the reduced acquisition time, resulting in a more extensive search of the parameter space of the depth and activity. In fact, this is clearly seen in Figure 12b; the joint distributions gradually diverged to neighboring depths and activities with decreasing acquisition time. It is worth mentioning that 10-s acquisition times are extremely short for a typical in situ measurements, which can lead to a highly fluctuating spectrum, as reported in Figure 12a.

### 3.4. Effect of the Gain Shift

**Figure 13:**
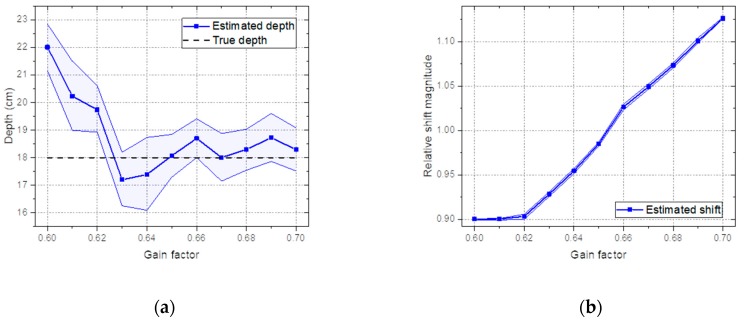
(**a**) Estimated depth and (**b**) estimated shift analyzed for the spectra of Cs-137obtained by changing the gain factor of the amplifier between 0.60 and 0.70 with 0.01 intervals.

**Figure 14:**
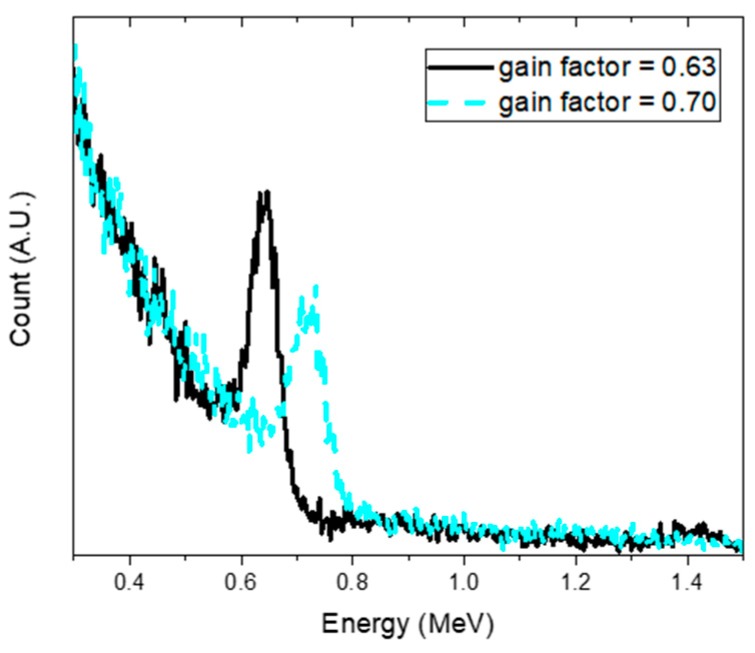
Example of the spectra of Cs-137 obtained with a gain factor of 0.63 (black solid line) and 0.70 (gray dashed line).

To demonstrate the ability to accommodate shifts in the spectra mainly due to temperature variations in the proposed model, spectra were measured with the Cs-137 source buried at a depth of 18 cm with the gain factor adjusted from 0.60 to 0.70 with 0.01 increments. Note that the test spectra were acquired with a gain factor of 0.65. In Figure 13a, we can see that the estimated depth fluctuated only slightly near the true depth for the spectra obtained with gain factors between 0.63 and 0.70. Furthermore, the true value of the depth fell within a 95% credible interval of the estimated depth within the investigated range. As expected, the trend of the change in the estimated shift increased with the increasing gain factor, as shown in Figure 13b. This was mainly because the model, combined with the bilinear interpolation method, scanned the shifted spectrum and searched for the probability distributions of the depth and shift that were most likely to have generated the spectrum via Bayesian inference. However, the estimated depth tended to increase and was thereby overestimated below the gain factor of 0.63. This was likely due to the slope connecting the Compton continuum and Compton valley in the spectrum becoming increasingly steep as the spectrum shifted in a negative direction, which is a typical phenomenon occurring when more gamma rays are scattered in a substance. Figure 14 shows an example of the spectra measured with gain factors of 0.63 and 0.70. It should be noted that these shifted spectra would be difficult to analyze without any recalibration.

### 3.5. Sensitivity of Prior Distributions

**Figure 15:**
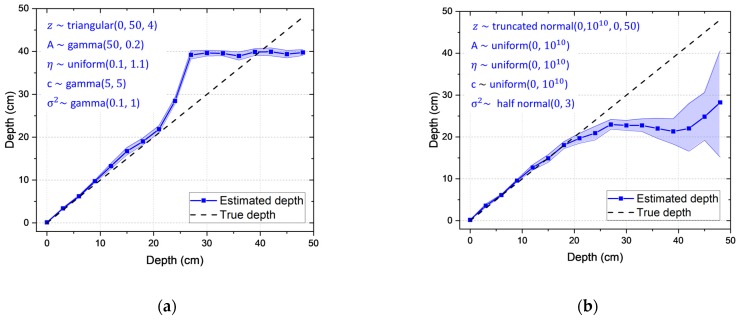
Estimated depths with a 95% credible interval for measured spectra for a Cs-137 source buried in sand from 0 to 48 cm with 3-cm intervals. These results were obtained with the prior distributions as reported within each figure. The letters marked in blue are the prior distributions that account for different beliefs.

To verify the robustness of our model with respect to the sensitivity to the prior distributions, experimental spectra for the Cs-137 source buried in sand from 0 to 48 cm with 3-cm intervals were analyzed using different prior distributions. Figure 15a shows the results using the following prior distributions: z followed a triangular distribution with parameters (0, 50, 4); A followed a gamma distribution with parameters (50, 0.2); η followed a uniform distribution with parameters (0.1, 1.1); c followed a gamma distribution with parameters (5, 5); and $\sigma^{2}$ followed a gamma distribution with parameters (0.1, 1). In other words, evaluators believed that the activity of Cs-137 source could be at a high level (i.e., the mean value was equal to 250 μCi) and buried deep (i.e., the mean value was equal to 18 cm). In addition, the observed spectrum could be shifted negatively. On the other hand, Figure 15b shows the results using the following prior distributions: A, η, and c all followed uniform distributions with parameters (0, 10^10^); z followed a truncated normal distribution with parameters (25, 10^10^, 0, 50) supported by z∈ [0, 50]; $\sigma^{2}$ followed a half-normal distribution with parameters (0, 3) supported by $\sigma^{2}\in$ [0, ∞]. Such a wide range of distribution can be non-informative for the data on a small numerical scale. In other words, evaluators would have very limited information. Thus, we confirmed that the same trends were observed up until the maximum detectable depth (i.e., 21 cm), as seen from the Cs-137 case (see Figure 7a), regardless of their different beliefs; the estimated depth agreed well with the true depth up to 21 cm. It is worth emphasizing that the proposed model may not be completely free from the choice on the prior distributions. Nevertheless, evaluators with some knowledge of radiation measurements may have a minimal influence over the posterior distributions.

## 4. Discussion

We presented an application of Bayesian inference to improve the estimation of remote depth profiling for low-level radioactive contaminants. From the simulation and experimental results, we confirmed that the proposed technique has significant advantages compared to existing methods for localized radioactive wastes. First, our approach allowed us to determine the probability distribution for parameters of interest, i.e., depth, activity, and shift, with improved reliability in a single measurement. From the measurement perspective, inherent uncertainty due to quantum physics is inevitable and must therefore be quantified. Thus, we should be able to provide measurement analysis in a statistical manner. However, previous depth estimation methods calculate uncertainty only via tedious repetitive measurements. Second, the proposed model yields a larger value for the maximum detectable depth. Recent studies have shown that the maximum detectable depths of 8.89-μCi Cs-137 and 0.24-μCi Co-60 buried in sand are 12 cm and 3 cm, respectively [14,15,16]. In fact, the activity intensity of the Cs-137 used in this work was about 10 times weaker than that of the Cs-137 used in those studies. Hence, the maximum detectable depth of 21 cm for both weakly active 0.94-μCi Cs-137 and 0.69-μCi Co-60 sources buried in sand was indeed a significant improvement in comparison to existing methods [9,10,11,12,13,14,15,16]. Third, the proposed technique provided a much faster and more accurate estimation of depths up to the maximum detectable depth (i.e., 21 cm), which was achieved within 60 s, even for sources with a weak activity. This is advantageous because radiological characterization for decommissioning involves scanning or measuring a wide range of sites, given that the activity of radioactive contaminants is not high enough in general. Fourth, the proposed technique was less susceptible to the gain shifts caused by temperature changes. One of the challenging issues for in situ measurement systems is that detectors are sensitive to changes in the ambient temperature, which can cause gain shifts. Therefore, regular quality control measurements become more critical to ensure a stable system operation. Thus, the present method automatically calibrated the degree of shift in the spectrum that would have been affected within the range of the prior distribution (see Table 1), which incorporated the magnitude of shifts in spectra due to temperature variation in the NaI(Tl) detectors [22]. In addition, the output of the shift could be used as a real-time indicator to demonstrate how stable the measurement systems are in operations on site. Finally, we confirmed that our model was not very sensitive to the choice of the prior distributions such that evaluators with some knowledge of radiation measurements would be able to obtain similar results. This feature is important for many practical applications because a Bayesian model is said to be non-robust and sensitive depending on the prior distributions.

However, this technique has some difficulties. One of the challenges in applying this technique lies in the establishment of a response matrix for materials in which a certain radioactive source is buried. To do this, the setup of materials affecting the attenuation of gamma rays should be carefully performed in order to mimic a real environment. Another difficulty is that the detector cannot simply be located at an optimal position in which the radioactive contaminants are buried; however, this can be resolved by placing the detector at the position in which the maximum intensity of the total count rate would likely be observed by taking a uniform scanning time. Lastly, most remote depth estimation methods assume that only a single radioisotope exists and that no other radioisotopes interfere with the measurement. In practice, such assumptions are sometimes not applicable. Furthermore, foreknowledge of radioisotopes present at a site is not available in some cases. Therefore, a better solution would be to integrate the likelihood function for quantitative analysis of radioisotopes, as proposed in Kim et al. [26] with the likelihood function used in this particular work. This solution will enable an accurate depth estimation for multiple radioactive sources without foreknowledge of the radioisotopes present.

## 5. Conclusions

In this work, we demonstrated an advanced method for remote depth estimation of localized radioactive contaminants using Bayesian inference. This approach, which is completely different from frequentist inference, allowed us to estimate the uncertainty of the depth and activity via a single measurement. The results of the simulation and experiment for Cs-137 and Co-60 sources buried in sand showed a significant improvement in the maximum detectable depth compared to those of existing methods. In addition, experimental results confirmed that the level of accuracy and the depth limit were preserved, even with a short acquisition time. Furthermore, the proposed technique was capable of accommodating for gain-shift effects caused by temperature variations, enabling a rapid non-intrusive localization of buried radioactive contaminants during in situ measurements as a consequence.

## Figures and Tables

**Table 1:** The priors prescribed in the model.

Variable	Prior
*z*	uniform(0, D)
*A*	gamma(1, 1)
η	uniform(0.85, 1.15)
*c*	gamma(1, 1)
$\;\sigma^{2}\;$	gamma(1, 1)

## References

[1] Characterization of Radioactively Contaminated Sites for Remediation Purposes. https://www-pub.iaea.org/MTCD/publications/PDF/te_1017_prn.pdf.

[2] Radiological Characterisation for Decommissioning of Nuclear Installations. https://www.oecd-nea.org/rwm/docs/2013/rwm-wpdd2013-2.pdf.

[3] Multi-Agency Radiation Survey and Site Investigation Manual (MARSSIM). https://www.nrc.gov/reading-rm/doc-collections/nuregs/staff/sr1575/r1/.

[4] Sullivan P.O., Nokhamzon J.G., Cantrel E. (2010). Decontamination and dismantling of radioactive concrete structures. NEA News.

[5] Dounreay Particles Advisory Group. https://assets.publishing.service.gov.uk/government/uploads/system/uploads/attachment_data/file/696380/DPAG_3rd__Report_September_2006.pdf.

[6] Dennis F., Morgan G., Henderson F. (2007). Dounreay hot particles: The story so far. J. Radiol. Prot..

[7] Popp A., Ardouin C., Alexander M., Blackley R., Murray A. Improvement of a high risk category source buried in the grounds of a hospital in cambodia. Proceedings of the 3th International Congress of the International Radiation Protection Association.

[8] Maeda K., Sasaki S., Kumai M., Sato I., Suto M., Ohsaka M., Goto T., Sakai H., Chigira T., Murata H. (2014). Distribution of radioactive nuclides of boring core samples extracted from concrete structures of reactor buildings in the fukushima daiichi nuclear power plant. J. Nucl. Sci. Technol..

[9] Shippen B.A., Joyce M.J. (2011). Extension of the linear depth attenuation method for the radioactivity depth analysis tool(RADPAT). IEEE Trans. Nucl. Sci..

[10] Shippen A., Joyce M.J. (2010). Profiling the depth of caesium-137 contamination in concrete via a relative linear attenuation model. Appl. Radiat. Isot..

[11] Adams J.C., Joyce M.J., Mellor M. (2012). The advancement of a technique using principal component analysis for the non-intrusive depth profiling of radioactive contamination. IEEE Trans. Nucl. Sci..

[12] Adams J.C., Joyce M.J., Mellor M. (2012). Depth profiling 137Cs and 60Co non-intrusively for a suite of industrial shielding materials and at depths beyond 50mm. Appl. Radiat. Isot..

[13] Adams J.C., Mellor M., Joyce M.J. (2011). Determination of the depth of localized radioactive contamination by 137Cs and 60Co in sand with principal component analysis. Environ. Sci. Technol..

[14] Ukaegbu I.K., Gamage K.A.A. (2018). A novel method for remote depth estimation of buried radioactive contamination. Sensors.

[15] Ukaegbu I.K., Gamage K.A.A. (2018). A model for remote depth estimation of buried radioactive wastes using CdZnTe detector. Sensors.

[16] Ukaegbu I.K., Gamage K.A.A., Aspinall M.D. (2019). Nonintrusive depth estimation of buried radioactive wastes using ground penetrating radar and a gamma ray detector. Remote Sens..

[17] Knoll G. (2010). Radiation interactions. Radiation Detection and Measurement.

[18] Wagenmakers E.-J., Lee M., Lodewyckx T., Iverson G.J. (2008). Bayesian versus frequentist inference. Bayesian Evaluation of Informative Hypotheses.

[19] Bishop C. (2006). Pattern Recognition and Machine Learning.

[20] Kucukelbir A., Tran D., Gelman A., Blei D.M. (2017). Automatic differentiation variational inference. J. Mach. Learn. Res..

[21] Salvatier J., Wiecki T.V., Fonnesbeck C. (2016). Probabilistic programming in python using PyMC3. PeerJ Comput. Sci..

[22] Kim J., Lim K.T., Kim J., Kim C., Jeon B. (2019). Quantitative analysis of NaI (Tl) gamma-ray spectrometry using an artificial neural network. Nucl. Instrum. Methods Phys. Res. Sect. A Accel. Spectrometers Detect. Assoc. Equip..

[23] Goorley J.T., James M.R., Booth T.E., Brown F.B., Bull J.S., Cox L.J., Durkee J.W., Elson J.S., Fensin M.L., Forster R.A. Initial MCNP6 Release Overview—MCNP6 version 1.0. https://permalink.lanl.gov/object/tr?what=info:lanl-repo/lareport/LA-UR-13-22934.

[24] Customs U.S., Protection B., Nuclear D., Office D. (2011). Compendium of Material Composition Data for Radiation Transport Modeling.

[25] Jeon B., Kim J., Moon M., Cho G. (2019). Parametric optimization for energy calibration and gamma response function of plastic scintillation detectors using a genetic algorithm. Nucl. Instrum. Methods Phys. Res. Sect. A Accel. Spectrometers Detect. Assoc. Equip..

[26] Kim J., Lim K.T., Kim J., Kim Y., Kim H. (2019). Quantification and uncertainty analysis of low-resolution gamma-ray spectrometry using Bayesian inference. Nucl. Instrum. Methods Phys. Res. Sect. A Accel. Spectrometers Detect. Assoc. Equip..

